# Impact of polar body biopsy on embryo morphokinetics—back to the roots in preimplantation genetic testing?

**DOI:** 10.1007/s10815-018-1207-4

**Published:** 2018-05-22

**Authors:** Michael Schenk, Andrea Groselj-Strele, Katharina Eberhard, Elisabeth Feldmeier, Darja Kastelic, Stefanie Cerk, Gregor Weiss

**Affiliations:** 1 Das Kinderwunsch Institut Schenk GmbH, Am Sendergrund 11, 8143 Dobl, Austria; 2 Institute of Human Genetics, Medical University of Graz, Neue Stiftingtalstrasse 2, 8010 Graz, Austria; 3 Core Facility Computational Bioanalytics, Center for Medical Research, Medical University of Graz, Stiftingtalstraße 24, 8010 Graz, Austria

**Keywords:** Preimplantation genetic testing, Polar body biopsy, Morphokinetic parameters, Time-lapse technology

## Abstract

**Purpose:**

Polar body biopsy (PBB) is a common technique in preimplantation genetic testing (PGT) to assess the chromosomal status of the oocyte. Numerous studies have been implemented to investigate the impact of biopsies on embryo development; however, information on embryo morphokinetics is still lacking. Hence, we investigated the impact of PBB on morphokinetic parameters in early embryo development.

**Methods:**

Four hundred four embryos (202 PBB, 202 control) were retrospectively analyzed. Patients were stimulated with a gonadotropin-releasing hormone antagonist ovarian hyperstimulation protocol. After fertilization check, embryos were incubated in a time-lapse incubator. The groups were matched for maternal age at time of oocyte retrieval.

**Results:**

Mean group times for reaching specific developmental time points showed no significant difference comparing embryos with PBB conducted and without. Likewise, further subdivision of the PBB group in euploid and aneuploid embryos revealed no differences in the early embryo morphokinetic development compared to the control group. Aneuploidy testing revealed a high prevalence of chromosomal aberrations for chromosomes 21, 4, 16, and 19.

**Conclusions:**

In conclusion, PBB does not impact the morphokinetic parameters of the embryo development. PBB can be safely applied without the risk of impairing the reproductive potential of the embryo and can be highly recommended as safe and practicable PGT approach, especially in countries with prevailing restrictions regarding PGT analysis.

## Introduction

Preimplantation genetic testing (PGT) has become a potent tool in assisted reproduction techniques (ART) within the last decades [1]. Among the different methods, polar body biopsy (PBB) has evolved to a common and secure method for infertile couples to assess the chromosomal status of the oocyte. Since polar bodies (PBs) are by-products of the meiotic division of the oocyte and are not required for fertilization and embryo development, they can be removed and subsequently screened for chromosomal aneuploidies without harming the embryo integrity [2]. The technique is less invasive than blastomere biopsy or trophectoderm (TE) biopsy and avoids false-positive errors due to the appearance of mosaicism, which is not present at the zygote stage [3]. PBB provides maternal genetic information exclusively; however, 90% of human aneuploidies at birth are of maternal origin [4]. In countries with legal restrictions regarding embryo biopsy like Austria or Switzerland, PBB diagnosis remains the only option for the investigation of chromosomal aneuploidy in oocytes in the first instance. Furthermore, PBB can be an alternative option for patients who want to avoid blastomere or TE biopsy due to ethical reasons.

Together with genetic analysis, time-lapse technology represents an efficient method to complement the embryo selection, essential to improve pregnancy rates [5]. Using time-lapse systems, morphokinetic parameters of the developing embryo can be observed accurately while environmental influences like temperature or humidity are minimized compared to traditional manual observation and embryo selection methods [6]. Back in 2011, the ALPHA Scientists in Reproductive Medicine and ESHRE Special Interest Group of Embryology elaborated a consensus for a standardized validation of kinetic parameters for embryo development [7]. Ciray et al. developed and proposed guidelines on the nomenclature and annotation of dynamic human embryo monitoring by time-lapse for the consensus [8]. This standardized evaluation helps to compare and unify embryo selection criteria between different in vitro fertilization (IVF) institutions. While the impact of biopsies on embryo development has been controversially discussed, information on embryo morphokinetic parameters in line with PBB is still lacking. Thus, the aim of this study was to investigate the effect of PBB on early embryo development to evaluate the potential risk of damaging oocyte integrity as well as the developmental potential of the embryo after PBB. Furthermore, differences in embryo transfer rates, biochemical pregnancy rates, implantation events, and the distribution pattern of genetic aberrations of aneuploid embryos were investigated.

## Material and methods

### Patients

**Fig. 1:**
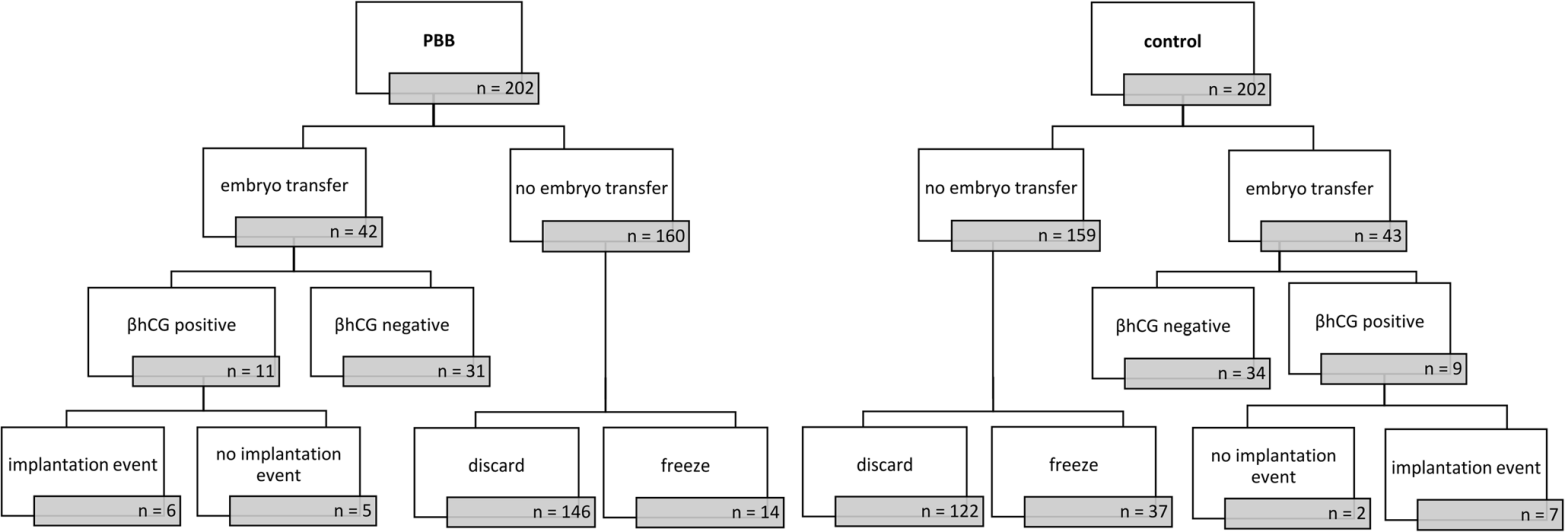
Flow chart showing the embryo outcome, selection, and quantity. The embryos were divided in one group with PBB conducted and one without (= control). A follow-up on transferred embryos was done by examining β-hCG test and implantation events. Embryos with no transfer were either discarded or cryopreserved according to embryonal development (both groups) and the genetic status (PBB group). The treatment procedure is illustrated top down

Four hundred four embryos from 79 female patients suffering from unexplained infertility, age 28–45 years, undergoing intracytoplasmic sperm injection (ICSI) treatment were retrospectively analyzed. Patients were excluded if they met the following criteria: (1) obesity (BMI > 30), (2) anorexia (BMI < 17.5), (3) endocrine disorders (including PCOS, reduced ovarian reserve defined by the Bologna criteria [9], premature menopause, hypothalamic amenorrhea, congenital adrenal hyperplasia), (4) diabetes mellitus, and (5) chronic inflammation. Additionally, sperm with paternal congenital disease or malformation was excluded. Indications for PBB were (a) increased maternal age, (b) known numerical or structural chromosomal aberrations, (c) implantation failure (three or more), or (d) recurrent miscarriage. The samples were classified in oocytes with PBB conducted (“PBB”; 38 women with 202 embryos) and not conducted (“control”; 41 women with 202 embryos). Patients in the control group also had indications for PBB; however, they refused the PBB analysis due to ethical or personal reasons. The groups were matched for maternal age at time of oocyte retrieval, since PBB is most commonly used with advanced maternal age and a concomitant reduced oocyte quality [10]. After PBB analysis, only euploid embryos were selected for transfer. The age distribution in both groups is identical, by month, to assure comparability between the two groups. Outcome of treatment procedure is displayed in Fig. 1. Data was collected from the IVF institution “Das Kinderwunsch Institut Schenk GmbH” in Dobl, Austria, from September 2013 to June 2015. An informed consent was obtained from each woman. The study was approved by the ethical committee of the Medical University of Graz, Austria (approval number: 20-492 ex08/09).

### Ovarian stimulation protocol

All women included in the study underwent GnRH antagonist protocol controlled ovarian hyperstimulation. Patients received recombinant human follicle-stimulating hormone (Puregon; MSD Sharp & Dohme GMBH) for 5 days with dosage adaption according to age, weight, sAMH concentration, and hormonal status [11, 12]. Trans-vaginal sonography was performed after 5 days of stimulation and on the day of oocyte retrieval. Ultrasonographical measurement was performed using a RIC 5-9-D 4D intravaginal probe of a GE Voluson E8 BT09 ultrasound machine (both from GE Healthcare Austria GmbH). GnRH antagonist (Cetrotide, Merck KGaA) was injected to avoid premature ovulation. Triggering was initiated 35 h before oocyte retrieval, administered with 5000–10,000 IU human chorionic gonadotropin (hCG) subcutaneously (Pregnyl, N.V. Organon), with dosage adaption according to body weight of the patient [11].

### Oocyte retrieval and fertilization

Oocyte retrieval was performed as previously described by Schenk and coworkers [13]. Briefly, follicles larger than 10 mm in diameter were aspirated and flushed (Flushing medium GM501 Flush; Gynemed Medizinprodukte GmbH & Co. KG) under sedation (Propofol, Fresenius Kabi Austria GmbH; Rapifen, Janssen-Cilag Pharma GmbH) and transvaginal ultrasound guidance (GE Healthcare Austria GmbH) with a Steiner-Tan needle 17 gauge and a Steiner flush/valve (IVFETFLEX.com, HandelsgmbH & Co KG). Follicular fluid (FF) and flushing volumes were examined for oocytes under constant conditions of 37 °C in an IVF workstation L24E with heating stage (K-SYSTEMS Kivex Biotec A/S). ICSI was performed on all MII oocytes 4–5 h after oocyte retrieval according to our standard operating procedure in both groups of patients. The method of collection and storage of FF as well as other body liquids within the frame of IVF (blood serum, cumulus cells, seminal plasma, embryo culture supernatant) was previously described by Schenk et al. [14, 15].

### Time-lapse incubation and embryo analysis

After oocyte retrieval and fertilization, oocytes were cultivated in universal culture medium (Gynemed Medizinprodukte GmbH & Co. KG, Germany) in a Forma CO_2_ incubator (Thermo Fisher Scientific, USA). After 14–16 h, fertilization check was performed. All normal fertilized embryos with two pronuclei (PN) were then cultured using Embryoslide dishes in Embryoscope® time-lapse incubator (both Vitrolife AB, Sweden) with 21% oxygen concentration. In the PBB conducted group, zygotes were transferred into the Embryoscope after biopsy. With the built-in camera and microscope, images of the developing embryo were taken every 15 min in seven different layers. Definition of morphokinetic parameters was performed according to the criteria proposed by Ciray et al. [8] (Table 1) and was analyzed with software developed for time-lapse image analysis (Embryoviewer® software; Vitrolife AB, Sweden).

**Table 1:** Morphokinetic variables and proposed definitions adapted from Ciray et al. (8)

Time	Definition of expected events
t0	Time of IVF or mid-time of micro/injection (ICSI/IMSI)
tPN	Fertilization status is confirmed
tPNf	Time of pronuclei disappearance; tPN1f; tPN2f
t2 to t9	2 to 9 discrete cells
tMor	End of compaction process (last frame before cavity formation)

### Polar body biopsy

Both first and second polar bodies were simultaneously biopsied on all zygotes with two PN 14–16 h after ICSI. PBs were biopsied in HEPES-buffered medium (Gynemed GmbH & Co. KG, Germany). The zygote was rotated and fixed to a 9-o’clock position using a holding pipette (5 μm in diameter) (Microtech IVF S.r.o., Czech Republic) and PBs’ position was at 11 o’clock. The zona pellucida was opened using a laser shot system (Octax Microscience GmbH, Germany) near the PBs which were aspirated with a biopsy pipette (Microtech IVF S.r.o.) and subsequently transferred into a 0.2-ml PCR tube (vWR International GmbH, Germany) filled with 4.5 μl nuclease-free water (Promega GmbH, Germany). PBs were stored at 2–8 °C until comparative genomic hybridization (CGH) analysis.

### Whole genome amplification and array CGH

Genetic analysis via whole genome amplification and CGH was performed by Single Cell Dimensions Genetics for Life GmbH, Graz, Austria, according to the manufacturer’s protocols. Results were provided within 48 h.

### Embryo transfer

After transabdominal ultrasound guidance (GE Healthcare Austria GmbH), a maximum of two euploid embryos were transferred on day 2, 3, 4, or 5 using an embryo transfer catheter set (Labotect Labor Technik Göttingen GmbH, Germany). The Istanbul consensus by the Alpha Executive and ESHRE Special Interest Group of Embryology [7] was used as morphology assessment criteria to select best viable embryos. The decision for the day of transfer was dependent on the embryo development, number of embryos, and the day when the genetic analysis was received. Embryos with PBB are usually transferred on day 4 to avoid premature blastocyst hatching. Double embryo transfer was performed seven times in the PBB group and ten times in the control group on explicit request of the patients. If more than one embryo was suitable for transfer and no double ET was requested, remaining embryos were cryopreserved using the Kitazato vitrification system (Kitazato Corporation, Japan). There is no follow-up on the outcome of the frozen embryos since only fresh cycles were used for this study.

### Statistical analysis

Continuous variables were reported by means ± standard deviations (SD), whereas count data were summarized using absolute frequencies and percentages. Comparisons between groups were done for categorical data by using the chi-square test or Fisher exact test. Continuous variables were examined for normality by the Kolmogorov-Smirnov test and the Shapiro-Wilk test with Lilliefors significance correction as well as by visual data inspection using Q-Q plots. Relationships between continuous variables were checked with Pearson’s correlation coefficients. Linear mixed effects models were performed to deal with random effects and with unequal sample sizes for time-lapse data. The linear mixed effects models were performed as restricted maximum likelihood (REML) approach [16]. Time in minutes to reach a specific developmental stage, measured with time-lapse technology, was the dependent variable in the model. Polar body biopsy conducted or not conducted was included as fixed between-group effect and developmental markers (tPNf, t2, t3, t4, t5, t6, t7, t8, tMor) as within-group effect. Interaction effects between biopsy and developmental markers were also considered in the model as well as the patient ID as person-specific random effect. A first-order autoregressive covariance structure was used for calculation of significant differences between groups and interactions. The model selection process to define the appropriate covariance structure of the repeated effect and the random effect was based on Akaike information criterion (AIC) and Bayesian information criterion (BIC), indices of relative goodness-of-fit for the linear mixed effects model, whereas the latter criterion takes the estimation of the covariance parameters more severely into account. A two-tailed *p* value of less than *p* < 0.05 was considered as statistically significant. All statistical tests were performed using SPSS version 23.0 (SPSS Inc., Chicago, IL) and GraphPad Prism version 6.05 (GraphPad Software, San Diego, USA) for visualizations.

## Results

### Patients’ characteristics

Female patients undergoing fertility treatment were 37.4 ± 3.39 years old and had a BMI of 23.7 ± 4.74 kg/m^2^. The number of oocytes obtained during oocyte retrieval varied between one and nine oocytes. A total of 404 embryos were analyzed.

Outcome of treatment procedure is displayed in Fig. 1. In the PBB group, 42 (20.8%) embryos were transferred compared to 43 (21.3%) embryos in the control group. From 85 transferred embryos, 6 were transferred on day 2 (control: 5; PBB: 1), 29 on day 3 (control: 20; PBB: 9), 37 on day 4 (control: 8; PBB: 29), and 13 on day 5 (control: 10; PBB: 3), according to the respective embryo quality and development. Transfer success was equal in both groups (*p* = 1.000). In the PBB group, 160 embryos were not transferred (146 discarded, 14 cryopreserved) compared to 159 embryos in the control group (122 discarded, 37 cryopreserved).

From the patients with a transferred embryo, 11 (26.2%) patients became pregnant confirmed by a positive beta hCG in the PBB group versus 9 (20.9%) patients in the control group. PBB did not influence chemical pregnancy rates (*p* = 0.798). In the PBB conducted group, 6 (14.3%) embryos successfully implanted and reached 12th week of gestation as compared with 7 (16.7%) embryos in the control group. All comparisons between groups revealed no significant results. Both groups reached equal pregnancy rates (*p* = 1.000).

### Embryo morphokinetics

The variable effects of group (PBB vs. control), developmental markers, and interaction terms to reach a specific developmental stage were evaluated. Time values were started to be measured 14–16 h after ICSI and after PBB in the time-lapse incubator. The time of pronuclei disappearance (tPNf) was the first parameter detected.

**Fig. 2:**
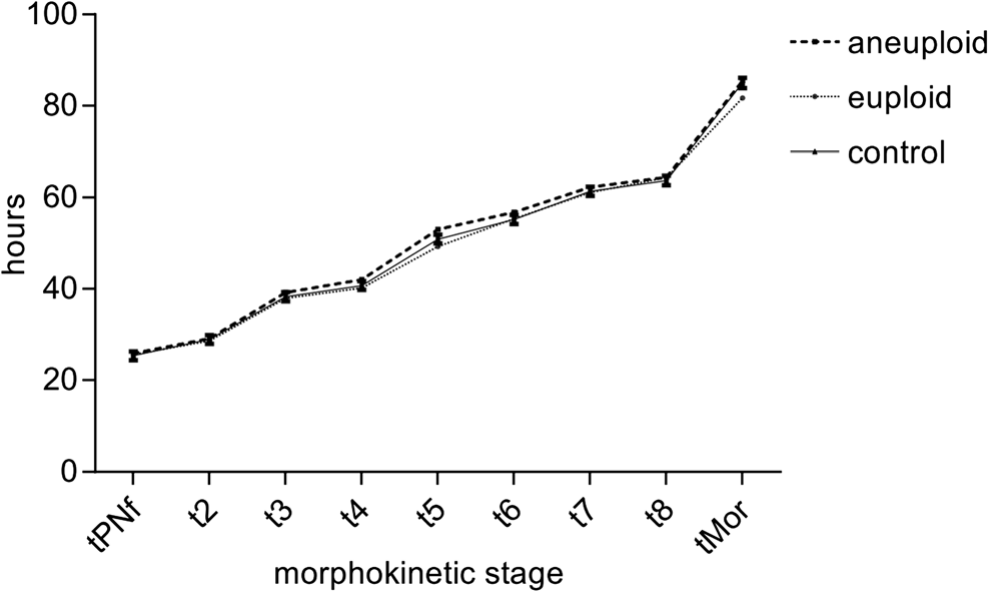
Group comparison for embryo developmental stages over time (h) for the groups “aneuploid,” “euploid,” and “control.” The time parameters of all three groups do not significantly differ and show almost identical values

In general, time in minutes to reach the developmental stages/markers tPNf, t2, t3, t4, t5, t6, t7, t8, and tMor, measured with time-lapse technology, differed significantly between the various time points (*p* < 0.001). However, there are no differences between biopsy conducted or control (main effect group: *p* = 0.964). Furthermore, mean group times for reaching tPNf, t2, t3, t4, t5, t6, t7, t8, and tMor showed no significant difference comparing PBB embryos and control (*p* = 0.872) (Table 2). Likewise, further subdivision of the PBB group in euploid and aneuploid embryos did not show any differences in reaching the developmental stages between aneuploid, euploid, and control group (*p* = 0.281) (Fig. 2, Table 3).

**Table 2:** Effect of group (biopsy conducted or not conducted), developmental markers (tPNf, t2, t3, t4, t5, t6, t7, t8, tMor), and interaction terms on time in minutes to reach a specific developmental stage, measured at time lapse

Variable	Numerator df	Denominator df	*F*	*p*
Intercept	1	10.326	4451.896	*p* < 0.001
Group	1	74.290	0.002	0.964
Developmental markers	8	2190.267	1034.572	*p* < 0.001
Group * developmental markers	8	2189.992	0.478	0.872

Linear mixed model; type III tests of fixed effects

**Table 3:** Effect of group (euploid, aneuploid, controls), developmental markers (tPNf, t2, t3, t4, t5, t6, t7, t8, tMor), and interaction terms on time in minutes to reach a specific developmental stage, measured at time lapse

Variable	Numerator df	Denominator df	*F*	*p*
Intercept	1	13.503	3822.022	*p* < 0.001
Group	2	211.466	1.218	0.298
Developmental markers	8	2025.748	715.666	*p* < 0.001
Group * developmental markers	16	2069.825	1.174	0.281

Linear mixed model; type III tests of fixed effects

### Analysis of chromosomal aberrations

**Fig. 3:**
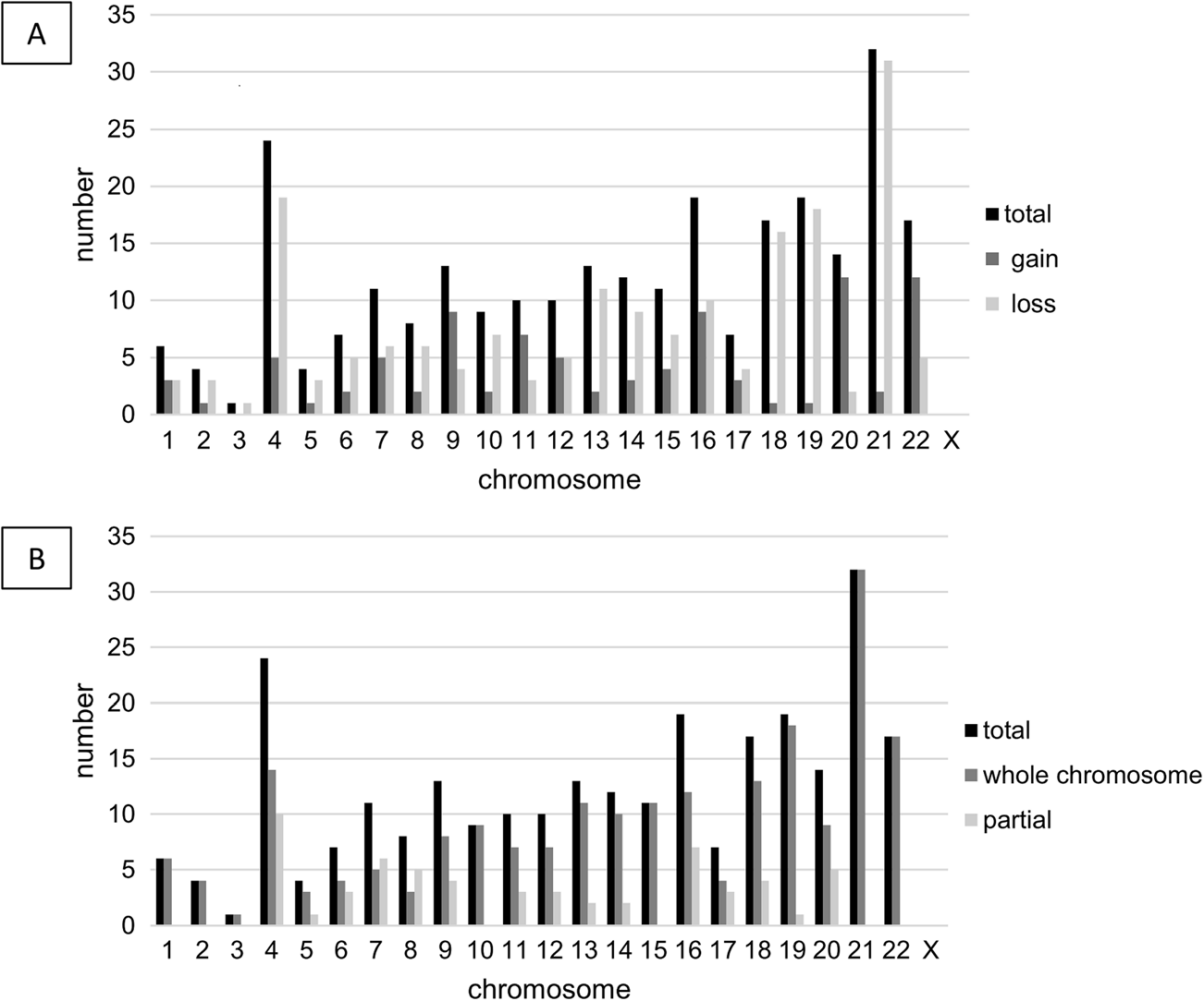
Aneuploidy screening. **a**) Distribution of chromosomes 1–22 and chromosome X, respectively, in respect to the individual gains and losses, resulting from the array CGH analysis. **b**) Distribution of chromosomal aberrations is displayed in respect to which part of the chromosome is concerned or if the whole chromosome is affected

Numerical as well as unbalanced structural chromosome aberrations were analyzed using array CGH. In total, 202 zygotes underwent PBB, from which 176 (87%) polar body biopsies were genetically tested and 26 (13%) could not be analyzed due to, e.g., insufficient DNA quality. From 176 PBB analyses, 44 (25%) were euploid, whereas 132 (75%) were aneuploid. Figure 3a shows the distribution of chromosomal aberrations (gains and losses) present in the tested polar bodies. Most alterations concerned chromosome 21, followed by chromosomes 4, 16, 19, 18, and 22. Predominant gains were found for chromosomes 9, 11, 20, and 22. By contrast, no aberrations were found for chromosome X. Slightly more losses than gains of chromosomal DNA were observed in chromosomes 4, 13, 18, 19, and 21.

Further analysis revealed that aberrations tend to concern the whole chromosome, but also partial alterations in individual chromosomes were observed (Fig. 3b). Single loss of chromosomes 21, 18, and 13 in the polar body—pointing to a corresponding trisomy in the oocyte—summed up to 11.36% of all aneuploid polar bodies.

## Discussion

Using modern time-lapse technology, the current study is the first one addressing the influence of PBB on morphokinetics of early embryo development. The data provided evidence that PBB does not affect achievement of the distinct morphokinetic stages tPNf, t2–t9, and tMor. Furthermore, we revealed that embryo transfer rates, biochemical pregnancy rates, and implantation events were not influenced by PBB and added knowledge about the distribution of chromosomal aberrations in aneuploid embryos.

The invention of PB-based aneuploidy screening in 1990 by Verlinsky et al. [17] offered the possibility to detect unbalanced structural and numeric chromosome aberrations and genetic diseases, respectively. Compared to embryo biopsy techniques like TE biopsy or blastomere biopsy, PBB is less invasive, since it only comprises the removal of PBs that display waste products of meiosis of the oocyte [2]. Furthermore, a fresh embryo transfer can be considered by using this technique, making it a valuable alternative compared to other embryo biopsy methods. However, safety and applicability of the PBB procedure are controversially discussed in the literature. To date, no sufficiently powered studies revealed a negative impact on embryo development after a PBB approach. According to the ESHRE Consortium, the practice of PBB has been controversial and dropped over the last years [18]; however, a proof-of-principle study by the ESHRE PGS task force indicated a ploidy prediction of oocytes with acceptable accuracy by array CGH analysis of both PBs [19]. Numerous researchers support PBB, especially in countries like Austria or Switzerland, where PBB is one of the only legal alternatives for genetical screening. Despite conflicting results, studies revealed that quality parameters, such as neonatal outcome [20] or embryo development [21], were not influenced by PBB. These findings are in line with our results, confirming the safety and practicability of the PBB approach. Additionally, our data suggest no differences in biochemical pregnancy rates, as measured by positive beta hCG, and implantation events (as measured by ultrasound), which further favors the application. Even though embryos selected by PBB are thought to improve pregnancy rates, the influence of maternal age on the successful outcome in fertility treatment can never be disregarded.

Genetic screening of the PBs exhibited that the majority (75%) of the tested oocytes contain an altered chromosome set. Aneuploidy is the leading genetic cause of human miscarriage and the majority result from nondisjunction events during maternal meiosis I [22]. Most aberrations—mainly losses—were detected concerning chromosome 21 within the PB, which point to a trisomic oocyte. This is consistent with the fact that trisomy 21 is the most common aneuploidy, followed by trisomy 18 [23]. In general, autosomal trisomies are not compatible with life and aneuploidies are responsible for around one-third of all pregnancy losses [22]. But there are few exceptions like trisomy 21, trisomy 18, and trisomy 13 that refer to Down’s syndrome, Patau’s syndrome, and Edwards’ syndrome, respectively [24]. Hence, they are of great clinical relevance. We detected the presence of these trisomies in 11.36% of all aneuploid oocytes, leading to the suggestion that PBB may be a powerful tool to prevent the occurrence of these birth defects. Furthermore, the possibility of detecting all chromosome aberrations makes PBB favorable to select the most promising oocytes, thereby avoiding embryo overproduction and assuring a transfer of a euploid embryo to the patient.

The impact on biopsy on human embryo developmental potential during PGT was reviewed by Cimadomo et al., favoring the blastocyst stage biopsy as alternative way of genetic testing [25]. Nowadays, TE biopsy is a commonly used method for aneuploidy screening, comprising both maternal and paternal origins of aberrations. However, it was recently shown that a single TE biopsy at blastocyst stage is statistically unable to determine embryo ploidy in an accurate way [26] and the incidence of mosaicism in preimplantation embryos is also reported up to 90% [27]. Furthermore, blastomere biopsy has been shown to delay embryo compaction and blastulation [28] in time-lapse monitoring. In respect to the presented results, PBB shows a solid alternative with low invasiveness, no mosaicism-related errors, safe applicability, and no harmful influence to the embryo development.

Morphokinetic parameters have evolved to become prominent targets for reproductive health research. In numerous studies, time-lapse technology revealed that timing of cell cycles and times between cell cycles are crucial steps in early embryonal development. Basile et al. identified that variables t3 (timing to three discrete cells) or t5 (timing to five discrete cells) are closely related to successful implantation [29]. Studies have demonstrated that morphokinetic variables can easily be influenced by detrimental disorders like the hyperandrogenic polycystic ovarian syndrome (PCOS), which was shown to cause a delay in t2, t3, t4, and t7 [30]. Additionally, aneuploid embryos exhibited a prolongation of t2 and t5 [31] and nicotine abuse caused delays in t3, t4, and t5 [32]. Interestingly, high-level mosaicism and structural aberrations are also common in good-quality embryos and are not restricted to arrested or poorly developing embryos [33]. Akarsu et al. [34] evaluated the impact of ovarian reserve and age on morphokinetic parameters and found tPNf, t2, t3, and t4 to be shorter in younger patients with normal ovarian reserve than in older patients. However, Gryshchenko et al. [35] could not find any morphokinetic differences between patients younger and older than 40 years. According to our data, PBB has no impact on the morphokinetic variables tPNf, t2–t9, and tMor, including the critical time parameters t3 and t5. Likewise, further subdivision of PBB embryo into euploid and aneuploid embryos did not show any differences in the embryonal development, assuming that the genetic status of the embryo does not influence early embryonal development as already confirmed by authors like Zhang et al. [36]. An interesting side effect of our study was the evaluation of laser beam influences on embryo development. Debates on the safety of the laser systems used in biopsy approaches are still ongoing with contradicting results [37, 38]. However, we provided evidence that the opening of the zona pellucida with laser beam had no influence on the morphokinetic parameters of early embryonal development.

Besides the advantages of PBB, we have to keep in mind that PBB analysis cannot predict mitotic errors during development leading to mosaicism later on; however, to date, there is no technique available which can exclude the problem of mosaicism completely. PBB can only evaluate the maternal contribution to a genetic disease while mitotic and paternal impacts remain unidentified. The majority of genetic aneuploidies have a maternal origin; however, a total of 10% of embryonic abnormalities is dedicated to paternal origin or post-fertilization contribution [39]. Maternal age is proposed as the main risk factor to predict a successful pregnancy [40], which was taken into account in the study design. The exact matching of the oocytes in respect to maternal age (month) at time of oocyte retrieval allows to reduce the influence of maternal age on morphokinetic development to a minimum, which must be considered the main strength of the study. Due to the small sample size of the study, the results must be interpreted with caution. Future studies should incorporate a larger data set to confirm the presented results.

## Conclusion

In conclusion, our results clearly showed that PBB does not impact the morphokinetic parameters of the embryo development and suggested no influence on embryo transfer, chemical pregnancy, and implantation events. Hence, PBB can be safely applied without the risk of impairing the reproductive potential of the embryo and can be highly recommended as safe and practicable approach to ensure transfer of euploid embryos for the patients, especially in countries with prevailing legal restrictions in PGT analysis. The current results raise the question if we have reached the gold standard in PGT analysis yet, since a risk in prediction and detection of genetic mosaicism obviously remains also in modern technologies like TE biopsy. It is tempting to speculate that PBB is about to regain attention for future studies of aneuploidy testing.
